# Disease Mechanisms and Therapeutic Approaches in SMARD1—Insights from Animal Models and Cell Models

**DOI:** 10.3390/biomedicines12040845

**Published:** 2024-04-11

**Authors:** Sibylle Jablonka, Ezgi Yildirim

**Affiliations:** Institute of Clinical Neurobiology, University Hospital Würzburg, Versbacher Strasse 5, 97078 Würzburg, Germany; Yildirim_E@ukw.de

**Keywords:** spinal muscular atrophy with respiratory distress type 1, SMARD1, motoneuron, muscle, helicase, IGHMBP2

## Abstract

Spinal muscular atrophy with respiratory distress type 1 (SMARD1) is a fatal childhood motoneuron disease caused by mutations in the *IGHMBP2* gene. It is characterized by muscle weakness, initially affecting the distal extremities due to the degeneration of spinal α-motoneurons, and respiratory distress, due to the paralysis of the diaphragm. Infantile forms with a severe course of the disease can be distinguished from juvenile forms with a milder course. Mutations in the *IGHMBP2* gene have also been found in patients with peripheral neuropathy Charcot–Marie–Tooth type 2S (CMT2S). IGHMBP2 is an ATP-dependent 5′→3′ RNA helicase thought to be involved in translational mechanisms. In recent years, several animal models representing both SMARD1 forms and CMT2S have been generated to initially study disease mechanisms. Later, the models showed very well that both stem cell therapies and the delivery of the human *IGHMBP2* cDNA by AAV9 approaches (AAV9-*IGHMBP2)* can lead to significant improvements in disease symptoms. Therefore, the SMARD1 animal models, in addition to the cellular models, provide an inexhaustible source for obtaining knowledge of disease mechanisms, disease progression at the cellular level, and deeper insights into the development of therapies against SMARD1.

## 1. Introduction

In recent years, the possibilities for treating monogenic disorders have increased significantly. One example of successful gene therapy is the classical form of spinal muscular atrophy (SMA). Using antisense oligonucleotides (ASOs), small molecules, or adeno-associated viruses 2.9 (AAV9), significantly increasing the levels of the disease gene, *Survival Motor Neuron* (*SMN*), in SMA patients has been possible. The improvement in the clinical picture was significant, especially when the therapies were used pre-symptomatically [1]. However, this development was only possible because of the extensive knowledge of genetics and cell and molecular biology using various cell and animal models, particularly the SMA mouse model. These insights can now be applied to other spinal muscular atrophies.

In this review, we will discuss the current state of research on disease mechanisms and therapeutic approaches in spinal muscular atrophy with respiratory distress type 1 (SMARD1). We will also discuss the implications of using SMARD1 mouse models for future cell and gene therapies. When discussing genetic approaches based on the delivery of the disease-causing *immunoglobulin helicase µ-binding protein 2* (*IGHMBP2*) gene by adeno-associated viruses (AAV), a distinction is always made between the human IGHMBP2 and the mouse Ighmbp2 protein.

### 1.1. Spinal Muscular Atrophy with Respiratory Distress Type 1 (SMARD1)

**Figure 1:**
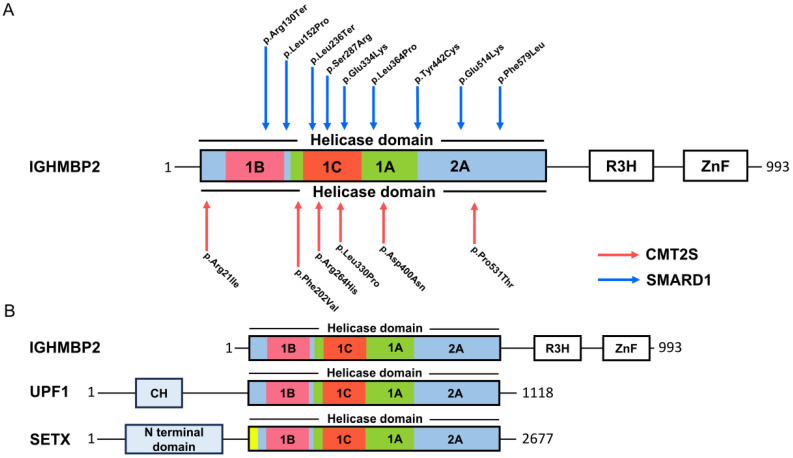
Mutations and protein domains in IGHMBP2. (**A**) Mutations (protein changes) in the helicase domain associated with SMARD1 and CMTS2. (**B**) Protein domain distribution in UPF1, SETX, and IGHMBP2.

SMARD1 is a spinal muscular atrophy with its own clinical entity. SMAs are characterized by spinal α-motoneuron loss during childhood with a fatal outcome. The loss of α-motoneurons results in the atrophy of skeletal muscles, which inevitably results in muscle paralysis. SMAs are monogenetic diseases and are caused by different disease genes. The disease gene of the proximal form of SMA is the *SMN* gene, which is located on chromosome 5q. SMARD1, also referred to as DSMA1 (distal spinal muscular atrophy type 1), was first described in 1974 under the assumption that it was SMA 5q. In 1996 it was accepted as a distinct clinical entity [2,3]. The current prevalence is unknown. The symptoms of SMARD1 are caused by mutations in the *IGHMBP2* gene (Figure 1A), which encodes for a 5′→3′ RNA helicase/ATPase [4] and ultimately results in the loss of helicase function [5]. This corresponds to dysfunction, degeneration, and loss of α-motoneurons in the ventral horn of the spinal cord, which ultimately leads to atrophy of skeletal muscle fibers in the extremities, trunk, and diaphragm [6]. Despite similar pathological signs, the disease is very distinct from the proximal form of SMA. First, a very rapid progression of muscle atrophy in the first few years of life starts with the distal limb muscles and then extends to the proximal regions. Second, a predominant clinical feature is the early onset of life-threatening respiratory distress due to the severe paralysis of the diaphragm caused by the degeneration of the phrenic nerve. This requires mechanical ventilation very early, between the first six weeks and up to six months of life [6,7,8]. In contrast, SMA patients develop respiratory distress in the later stages of the disease due to the paralysis of the intercostal muscles [9]. Respiratory distress is always associated with inspiratory stridor, weak crying, recurrent bronchopneumonia, and feeding difficulties, which are always life-threatening in the absence of medication [9]. SMARD1 leads to the paralysis of all extremities, often associated with an absence of deep tendon reflexes and rachis malformations, such as kyphoscoliosis, usually after the first year of life. Clinical symptoms often stabilize after the first two years of life, and sometimes there are slight improvements in respiratory activity and muscle strength [7,10,11]. Motor symptoms are often associated with progressive autonomic dysfunction [7]. Autonomic nervous system involvement most commonly manifests as bladder incontinence, urinary retention requiring catheterization, excessive sweating, constipation, and cardiac arrhythmias [7,11]. Two types of the disease develop according to the onset and severity of the disease: infantile and juvenile SMARD1 [8,11,12,13,14]. In infantile SMARD1 patients, no direct correlation between the mutation and the phenotype has been observed [7,12]. However, patients with juvenile SMARD1 show constant steady-state levels of the IGHMBP2 protein [14].

Recently, mutations in *IGHBMBP2* have been described in an axonal form of Charcot–Marie–Tooth (CMT), classified as CMT type 2S (CMT2S) [5,15,16,17,18,19,20,21] (Figure 1A). CMT2S is a sensorimotor axonal polyneuropathy with normal or near-normal nerve conduction velocity (NCV). Neurophysiological changes and respiratory defects in CMT2S are less pronounced compared to SMARD1. CMT2S is estimated to affect 1 in 2500 individuals with disease onset between 5 and 25 years, with longer survival compared to SMARD1 patients [22]. In 2014, Cottenie and colleagues combined genome sequencing and linkage analysis to identify mutations in the *IGHMBP2* gene in two consanguineous English CMT2S patients [16]. Mutations in the *IGHMBP2* gene in CMT2S patients tend to have a milder involvement of large, myelinated fibers. *IGHMBP2* mutations in SMARD1 patients are mainly missense mutations in the helicase domain. In contrast, in CMT2S, the mutations were primarily a combination of a nonsense mutation in the 5′ region of the gene, with truncating frameshift, missense, or homozygous frameshift mutations in the last exon [5]. Finally, different combinations of mutations in the two diseases resulted in different levels of residual protein. On average, CMT2S patients have a higher level of IGHMBP2 than SMARD1 patients [16]. Tian and colleagues have summarized the proportion of *IGHMBP2* gene variants in SMARD1 and CMT2S. Non-truncating mutations in the RecA-like domains (1A and 2A) are hotspots for SMARD1, whereas truncating mutations in the last exon are hotspots for CMT2S [5]. However, Tran and colleagues have recently identified an *IGHMBP2* mutation that leads to either SMARD1 or CMT2S, suggesting that the individual genetic background with potential disease-modifying regions plays a critical role in the expression of SMARD1 or CMTS2 [23].

### 1.2. Yeast Assay Analysis of Single IGHMBP2 Mutations

The molecular and cellular biological influence of single *IGHMBP2* mutations is not straightforward, as they are always compound heterozygous in SMARD1 and CMT2S patients. The development of relevant mouse and iPSC models in which the mutations are homozygous is time- and resource-consuming. To obtain certainty about the pathophysiology of single *IGHMBP2* mutations more quickly, Rzepnikowska and colleagues have developed a yeast model. IGHMBP2 is well conserved, and its homologs are found in many model organisms, from Saccharomyces cerevisiae to mice [24]. The group generated a mutant Saccharomyces cerevisiae homolog of IGHMBP2 (Hcs1) that does not express Hcs1 (hcs1Δ) and is highly sensitive to the translation blocker cycloheximide (CHX) in terms of cell division. By using a phenotype of CHX hypersensitivity, they analyzed human *IGHMBP2* variants by testing each mutation individually for its ability to rescue the hcs1Δ phenotype. The yeast system makes it possible to distinguish mutations from polymorphisms. The classification of gene variants is fundamental to genetic testing.

## 2. IGHMBP2 Function and Modifiers of IGHMBP2 Deficiency

**Figure 2:**
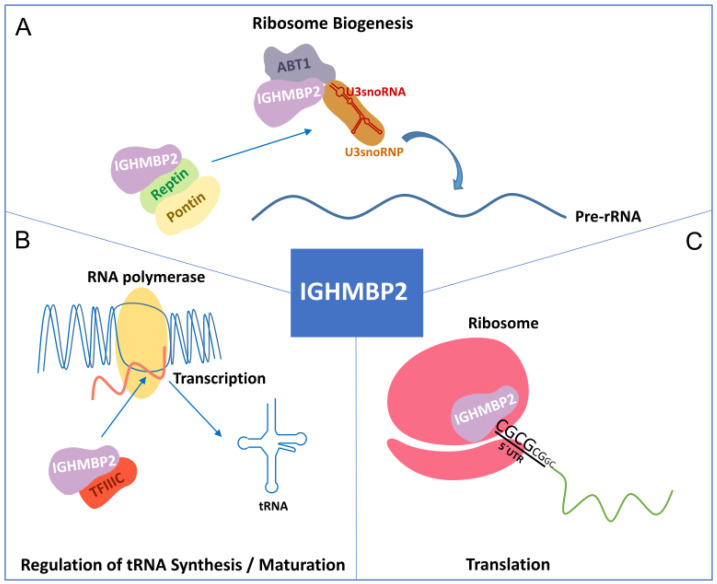
Function of IGHMBP2. (**A**) IGHMBP2 is associated with Reptin and Pontin. In addition, the IGHMBP2/ABT1 complex interacts with the 5′ external spacer of pre-rRNA, highlighting the involvement of IGHMBP2 in ribosome biogenesis. (**B**) IGHMBP2 binds to the 220 kDa transcription factor IIIC (TFIIIC220) for tRNA transcription. (**C**) IGHMBP2 interacts with the 80S ribosomal subunit and binds to the 5′UTR of GC-rich mRNAs.

Helicases are ubiquitous and highly conserved enzymes. They remodel nucleic acids or nucleic acid-protein complexes by ATP hydrolysis. Based on sequence, structure, and functionality, helicases are classified into six superfamilies (SFs) with many functions in different areas of DNA and RNA metabolism. SF1 and SF2 consist of non-ring-forming helicases, including all eukaryotic RNA and DNA helicases [25,26]. They use a highly conserved helicase core consisting of two tandem RecA-like domains, and they are characterized by 12 characteristic sequence motifs [26,27]. The helicase IGHMBP2 is a member of SF1 [25] and thus belongs to a group of UPF1-like helicases, such as UPF1, Senataxin (STX) (Figure 1B), and MOV10 [26,28]. IGHMBP2 consists of 993 amino acids [29] and is composed of a DexxQ-type helicase/ATPase domain, an R3H domain, and a zinc finger domain. The helicase core of IGHMBP2 contains four domains: two RecA-like domains and two subdomains [28]. As an ATP-dependent 5′→3 helicase, IGHMBP2 unpacks RNA and DNA duplices and co-localizes ribosomal subunits [30]. Co-localization is also observed with tRNAs or factors that are important for tRNA transcription and ribosome maturation, such as tRNATyr, TFIIIC220, proteins of large and small ribosomal subunit proteins, and 5S, 18S, and 28S rRNAs. IGHMBP2 also interacts with Reptin and Pontin, both of which play roles in U3 snoRNP biogenesis by interacting with U3 snoRNA [31]. The binding of IGHMBP2 to the activator of basal transcription (ABT1) in a complex by binding to the 5′ external transcribed spacer and the U3 snoRNA is important for pre-rRNA processing [32] (Figure 2A,B).

### 2.1. Modifiers of IGHMBP2 Deficiency

As early as 1998, Cox and colleagues were able to show that the severity of Ighmbp2 deficiency in mice depends on a so-called modifier region. A 166 kb CAST BAC clone (CH26-27k3) with 2 coding genes—*Abt1* and zinc finger protein (*Zfp322a*)—1 non-coding EST, and 24 tRNA genes was able to modify the mutant phenotype of a SMARD1 mouse model. It slowed the progression of motoneuron degeneration but did not restore *Ighmbp2* mRNA splicing or Ighmbp2 protein in the mouse to wild-type levels [31,33]. An association between ABT1 and IGHMBP2 was shown by co-immunoprecipitation when FLAG-IGHMBP2 and myc-ABT1 were co-transfected into HEK 293T cells [31,34]. Another study by Vadal and colleagues showed that the direct, high-affinity binding of ABT1 to IGHMBP2 increased ATPase and helicase activity and the processivity of IGHMBP2 [32].

### 2.2. Function of IGHMBP2

Ighmbp2 is predominantly localized in the cytoplasm of isolated mouse motoneurons but is less abundant in the nucleus [35,36]. Its exact function is unknown and its pathogenic role in SMARD1 is still unclear. However, a recent study by Prusty and colleagues clearly showed that IGHMBP2 is associated with polysomes and affects the translation of mRNAs with short GC-rich and structured 5′ UTRs to facilitate their ribosomal entry (Figure 2C). IGHMBP2 deficiency causes the ribosomal stalling of targeted mRNAs, leading to reduced translation. Some mRNA targets encode components of the THO complex, which is part of the transcription export (TREX) complex. This IGHMBP2-dependent regulation of the THO complex is detectable in induced astrocytes from SMARD1 patients [37].

Therefore, IGHMBP2 and its family members UPF1 and MOV10 are currently described as enzymes with regulatory functions in mRNA translation and/or metabolism [38,39]. A recent study by Kanaan and colleagues demonstrated the lower nucleic acid binding/unwinding capacity of IGHMBP2 in contrast to UPF1 [40]. The SF1 member UPF1 is involved in nonsense-mediated decay (NMD), causing degradation of mRNAs with coding sequence (CDS)-interrupting premature termination codons (PTCs) [38]. In addition to NMD, UPF1 has been identified as a helicase that is co-transcriptionally associated with mRNAs [41]. The RNA helicase activity of UPF1 is required for the release of mRNAs from transcription sites and their export from the nucleus. UPF1 constantly moves between the nucleus and the cytoplasm. One of the UPF1 candidates is *Actin5C* in Drosophila [41]. Strikingly, Ighmbp2 has also been reported to regulate β-actin proteins in the cell bodies and growth cones of primary cultured mouse motoneurons [36]. MOV10 functions as a messenger ribonucleoprotein (mRNP) clearance factor. It remodels the local secondary structures of mRNAs and displaces proteins from mRNAs which are targeted for UPF1-mediated degradation [39]. It remains to be seen whether IGHMBP2 has the same function in motoneurons. Taiana and colleagues have previously shown that *IGHMBP2* mRNA is regulated by NMD. Other mRNAs targeted by NMD are upregulated in SMARD1 iPSCs and induced motoneurons, and they were rescued via NMD reactivation [42].

An important aspect of elucidating the functional properties of RNA helicases is the identification of their mRNA or protein targets and their mode of action on these transcripts or proteins. Since most mutations (homozygous or compound heterozygous) are located within or close to the helicase domain, the helicase activity seems to be affected [14]. This requires the use of mouse models carrying either SMARD1 mutations with constant Ighmbp2 protein levels or mutations leading to Ighmbp2 deficiency with the corresponding tissue-specific phenotype.

## 3. Mouse Models of SMARD1 and Peripheral Neuropathies

The impaired cellular mechanisms that lead to SMARD1-specific motoneuron degeneration and CMT2S are far from being understood. Therefore, different mouse models have been created to understand the disease mechanisms that are summarized in Table 1.

### 3.1. The Nmd^2J^ Mouse as a Model for SMARD1

The neuromuscular degeneration mouse B6.BKS-*Ighmbp2^nmd−2J^*/J (*Nmd^2J^*) is a model for the juvenile form of SMARD1 (Table 1). The mouse carries a point mutation in the *Ighmbp2* gene [33]. This causes a reduction of 20–30% of the Ighmbp2 levels in all tissues [35] and primary cultured motoneurons [36]. Ighmbp2 is expressed at relatively high levels in the brain, spinal cord, and muscles [35]. Mutant *Ighmbp2* carries a single A to G transition 23 bp in intron 4 by creating a cryptic splice donor site. In all tissues, such as the liver, kidney, lung, heart, spleen, muscle, and thymus, the same level of abnormal splicing—from 20–25% wild-type splicing to 75–80% mutant splicing—has been observed as in the brain and spinal cord [33].

The pathological features of the *Nmd^2J^* mouse are comparable to those in humans. The loss of motoneurons (~30%) in *Nmd^2J^* mice is first detectable ten days after birth (P10), when these mice appear clinically unaffected with normal muscle strength. This means that the loss of motoneurons is an early event in the disease. No further reductions were observed until P21, when the first symptoms in the form of muscle weakness appeared but then progressed to the final stages [35,43]. Muscle weakness begins in the hind limbs and rapidly progresses to generalized limb and trunk muscle weakness between three and five weeks after birth. By P10, the *Nmd^2J^* mouse shows a loss of 37% of motoneuron cell bodies in the lumbar spinal cord, while 10% of motor axons in the femoral nerve begin to degenerate. This suggests that motoneuron degeneration begins at the level of the spinal cord and that the features of axonal degeneration are similar to those seen in Wallerian degeneration [35]. By 12–14 weeks, 70% of the motoneurons have degenerated, while 40% of the total number of axons in the femoral nerve (i.e., presumably 60–80% of the motor axons) are lost. This indicates motoneuron cell body loss in the lumbar spinal cord prior to axonopathy. Muscle fiber degeneration, including abundant myopathic changes in the diaphragm, begins around six to eight weeks after birth without motor axon loss in the phrenic nerve [35,43,44]. An additional role for Ighmbp2 in muscle fiber maintenance was discussed while in 14-week-old *Nmd^2J^* mice, where central nuclei and myofiber regeneration were prominent. In addition, and in contrast to SMARD1 patients, the *Nmd^2J^* mouse develops cardiomyopathy during the later stages of the disease [35,45]. According to Lee et al., the *Nmd^2J^* mouse has defects in bone quantity and architecture. The most severe defects were observed in the trabecular compartment [46]. The survival rate of the *Nmd^2J^* mouse is highly variable. It ranges from six weeks to eleven months, probably due to unknown modifying polymorphisms on chromosome 13 [33,47]. To prevent the modifying effect of the region on chromosome 13 of the C57Bl/6 mouse background, Shababi and colleagues generated a new mouse model: the FVB-*Nmd* mouse. The point mutation in intron 4 of the *Ighmbp2* gene on an FVB/NJ background (FVB/NJ-*Ighmbp2^nmd/nmd^*) shows a more severe phenotype in terms of survival, weight, and motor function [48]. Despite the now more stable survival rate, the new mouse model also showed no respiratory distress. In a recently published study, Vadla et al. introduced ABT1 as the first modifier of SMARD1 pathology. Through its association with IGHMBP2, ABT1 has a direct effect on disease severity by regulating the activities of IGHMBP2. Increasing ABT1 protein levels by the adeno-associated virus 2/9 (AAV9)-mediated delivery of scAAV9-*ABT1* to FVB/NJ-*Ighmbp2^nmd/nmd^* mice via intracerebroventricular (ICV) injection extends lifespan and counteracts neuromuscular junction (NMJ) denervation [32].

### 3.2. A SMARD1 Mouse with Respiratory Distress

To create a SMARD1 mouse that reflects the human disease phenotype in terms of respiratory distress, Smith et al. started to generate a new model on the FVB/NJ background [49] (Table 1). The D564N mutation identified in CMT2S patients was introduced into the *Ighmbp2* gene via CRISPR/Cas9. Homozygous *Ighmbp2*-D564N mutations in mice resulted in a respiratory phenotype in addition to motoneuron degeneration and decreased survival. The FVB/NJ-*Ighmbp2*^D564N^ homozygous mice showed reduced respiratory rates under normoxic conditions, and this was exacerbated under hypoxic and hypercapnic conditions. Surprisingly, despite the respiratory deficiency, the mouse model showed significant denervation in the muscle involved in limb movement, but not in the muscles involved in respiration [49], which is comparable to the *Nmd^2J^* mouse [4]. In both mouse models, the motor nerve that innervates the diaphragm does not appear to be affected. This may be because Ighmbp2 deficiency does not affect fibers from spinal cord segments C3 and C4, nor some parts of C5, but rather causes a myopathy of the diaphragm. These observations inevitably led to the conclusion that the myopathy of the diaphragm may also occur in humans, which must of course be considered in therapy.

### 3.3. Ighmbp2 Mouse Models Recapitulating Peripheral Neuropathies

*IGHMBP2* mutations in patients do not only exclusively lead to motoneuron loss; they also lead to peripheral neuropathies for reasons that are still unknown. Martin and colleagues were the first to characterize CMT2S mouse models with mutations in two different regions of the helicase [50] (Table 1). The E356del variant (C57BL/6J-Ighmbp2^em1Cx^/Cx) was introduced into the helicase domain via CRISPR/Cas, while the Y918C variant (C57BL/6J-Ighmbp2^em5Cx^/Cx), generated by a knock-in technique, was localized to the C-terminus, a domain that regulates RNA-binding affinities. *Ighmbp2*-E365del homozygous mice show reduced sensitivity to mechanical forces and decreased thermosensitivity and sensorimotor deficits. In terms of motor performance, mutant mice performed worse on the accelerating rotarod, and reduced endurance was measured in female mutants. These observations correspond to a progressive degeneration of myelinated sensory and motor axons of the femoral nerve between 10 and 20 weeks, including affected axonal calibers. In the gastrocnemius muscle, motor nerve terminals become progressively affected between 8 and 15 weeks of age, in that the presynaptic terminal is absent and the postsynaptic regions are empty. Since NMJ deficits outweigh motor axon loss, it is reasonable to assume that a dying-back mechanism is occurring. Y918C mice have a slightly more severe CMT2S phenotype than the E356del variant. They develop smaller hind limbs, smaller motor axon diameters, and approximately 30% fewer innervated NMJs at week 16, although the E356del variant develops more fiber loss on motor and sensory nerves without affecting nerve conduction velocity (NCV), unlike the Y918C variant.

Taken together, both models develop a less severe phenotype than the *Nmd^2J^* mouse, but this is similar to CMT2S patients without cardiac phenotypes and shortened lifespans [50]. Currently, the susceptibility to cardiomyopathy in SMARD1 patients is still an open question. This should be considered in the future using different mouse models in therapeutic development.

## 4. Insights into Cellular and Functional Dysregulation

### 4.1. Cellular Dysregulations in Motoneurons

The *Nmd^2J^* mouse is the best-studied model for cellular dysregulations caused by Ighmbp2 deficiency. Neuromuscular junctions in muscles in the *Nmd^2J^* model remain intact until the late stages of the disease, and neurotransmitter release appears relatively unaffected, as long as NMJs are maintained [43]. Villalon and colleagues showed that pathology includes NMJ changes but also defects in myelination [44]. These findings led to the conclusion that the loss of Ighmbp2-deficient motoneurons is not primarily due to neurotransmission failures at the NMJ [43]. Thus, the cellular dysregulations that lead to motoneuron degeneration under Ighmbp2 deficiency are currently not understood. Primary cultured motoneurons from *Nmd^2J^* mouse embryos only show minor morphological changes. Only a small increase in axonal branches on laminin-111 or longer axons on laminin-221 were seen [36]. Consistent with the rather mild phenotypic aberrations, only minor changes in the transcriptome (123 up- or downregulated transcripts in total) were identified by the RNA sequencing of Ighmbp2-deficient motoneurons. Similarly, but in contrast to observations in IGHMBP2-deficient non-neuronal cells [37], no global changes in protein synthesis were detected using pulsed SILAC (Stable Isotope Labeling by Amino acids in Cell culture), FUNCAT (FlUorescent Non-Canonical Amino acid Tagging), and SUnSET (SUrface SEnsing of Translation) approaches. Only a local reduction of the β-actin protein was observed in growth cones of Ighmbp2-deficient mouse motoneurons [36]. No impairment of Ca^2+^ homeostasis was measured with laminin-111, but using laminin-221 as a matrix protein reduced spontaneous Ca^2+^ transients, and impaired axon extension was detected [51]. These small cellular changes under Ighmbp2 deficiency in motoneurons suggest additional non-cell autonomous influences or suggest that only a small number of proteins/targets are responsible for the motoneuron loss in SMARD1.

### 4.2. Cellular Dysregulation Due to Reduced Growth Factor Release

**Figure 3:**
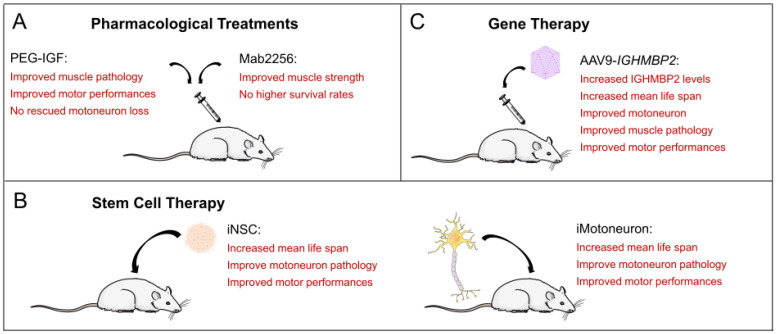
Potential treatment strategies for SMARD1. (**A**) Pharmacological treatments in the SMARD1 mouse. (**B**) Stem cell-derived treatments in the SMARD1 mouse. (**C**) AAV9-*IGHMBP2* therapy in the SMARD1 mouse.

Reduced caliber of gastrocnemius and diaphragm muscle fibers closely associated with impaired type I and type II fiber differentiation are the pathological features of muscle fiber degeneration in *Nmd^2J^* mice [35,43,44,52]. A first approach using a monoclonal antibody (Mab2256) with agonist activity against the NT-3 receptor TrkC produced transient improvements in muscle strength and normalized neuromuscular depression during high-frequency nerve stimulation. However, the survival rate of the *Nmd^2J^* mouse was still significantly reduced [53] (Figure 3A). A few years later, Krieger and colleagues investigated IGF1, another neurotrophic factor, which is involved in muscle and neuron survival and differentiation [52]. *Nmd^2J^* mice have an upregulation of the receptor for Igf1 (Igf1R) in the gastrocnemius muscle and the diaphragm, which is not observed in spinal cord tissue at P14 [52]. They were able to show nicely that *Nmd^2J^* mice not only exhibit Igf1 upregulation, but also reduced serum levels of Igf1. Strikingly, external application of human polyethylene glycol-coupled IGF1 (PEG-IGF1) was able to compensate for this deficiency [52]. This external application in the *Nmd^2J^* mouse resulted in a complete rescue of the muscle fiber type in the diaphragm and partially in the gastrocnemius muscle [52] (Figure 3A). Unfortunately, PEG-IGF1 does not compensate for spinal cord tissue [52]. Loss of motoneuron cell bodies and axons was still detectable in PEG-IGF1-treated *Nmd^2J^* mice. This raised the question of whether growth factor dysregulation primarily affects motoneuron development. Transplantation studies of wild-type iPSC-derived neural stem cells (NSCs) into *Nmd^2J^* mice showed neuroprotective effects through growth factor release [54]. Transplantation of neural stem cells increases the number of spinal motoneurons and prolongs the survival time of *Nmd^2J^* mice [55,56], which will be discussed in detail in the next chapter.

## 5. Stem Cell Therapy in SMARD1 Mouse Models

### 5.1. Transplantation of Mouse Stem Cells and Mouse Motoneurons

The first step towards stem cell therapy was taken some time ago by Corti and colleagues [55] (Figure 3B). It was known that motoneurons derived from embryonic stem (ESs) cells or spinal cord stem cells could be a possible therapeutic strategy for motor unit reconstitution, using retinoic acid (RA) and sonic hedgehog (SHH) for the differentiation process [57,58,59]. They first investigated the self-renewal and multipotential properties of aldehyde dehydrogenase (ALDH)-positive embryonic stem cells from the spinal cord of wild-type mice. Differentiation into motoneurons was induced by the addition of SHH, RA, nerve growth factor (NGF), and cAMP. The induced motoneurons (iMN) were then positive for ChAT, Islet1, and HB9. After finding that the iMN could form neuromuscular junctions, they were used for transplantation approaches via intrathecal application into *Nmd^2J^* mice. It was shown that after ALDH, stem cells were correctly differentiated into motoneurons in the spinal cord, and the transplanted *Nmd^2J^* mice did not exhibit typical hind limb posture even at three weeks after birth. At 5 weeks of age, they were still able to perform the rotarod test, which was at least better than the untreated animals. Transplanted *Nmd^2J^* mice showed an intermediate 30-day survival rate that was significantly higher and different from non-transplanted *Nmd^2J^* mice. In terms of motoneuron loss, transplanted *Nmd^2J^* mice exhibited a significant loss only after 6 weeks, accompanied by a moderate reduction in large L4–L5 axons in the ventral nerve roots (75% preservation) and de novo neurogenesis.

Corti and colleagues followed up this study three years later with another study showing that the direct transplantation of motoneurons into the spinal cord can rescue the motor phenotype in SMARD1 mice extremely well [60] (Figure 3B). They isolated neural stem cells (NSCs) from the ventricular zone (VZ) from the spinal cords of E10.5 HB9-GFP mouse embryos and differentiated them into motoneurons after induction with RA and SHH. Purified HB9-GFP-iMNs were transplanted into the spinal cord of 14-day-old *Nmd^2J^* mice. Some mice were also treated with cAMP, rolipram, and GDNF. Both transplanted *Nmd^2J^* colonies had increased body weight, significantly prolonged survival, and improved motor skills, as demonstrated by rotarod and hind limb clenching, comparable to control mice. Motor axons extended into the gray and the surrounding white matter. The HB9-GFP motoneurons co-expressed NF, MAP2, NeuN, and ChAT. GFP-labeled axons were detected in muscle tracts (biceps, triceps, quadriceps, tibialis anterior, and gastrocnemius muscles). Notably, the morphology of the newly generated neuromuscular junctions was like that of endogenous neuromuscular junctions. This second study impressively demonstrated that iMNs can also be used as grafts in motoneuron diseases. By improving the neurological phenotype, NSC transplantation can be discussed as a potential therapeutic strategy and may therefore be a general option for the treatment of neurodegenerative diseases. They can be used not only for neuroprotection, but also for replacement, as is already shown in the case of SMARD1 [55,60].

### 5.2. Transplantation of Human iPSC-Derived NSCs

Induced pluripotent stem cells (iPSCs) may be another option for therapeutic use (Figure 3B). Simone and colleagues transplanted iPSC-derived NSCs into the spinal cord of *Nmd^2J^* mice. iPSCs were converted into Sox1+2 and Nestin-positive self-renewing and multipotent neuroectodermal NSCs with TUJ1-positive processes [54]. The cells were then exposed to SHH, RA, and neurotrophic factors. The resulting neurons expressed ChAT, MAP2, Islet 1, and HB9. GFP-labeled iPSC-derived NSCs were transplanted into *Nmd^2J^* mice on day 1 (P1). The human donor cells exhibited neuronal and glial morphologies and expressed β-III tubulin, NeuN, Nestin, and GFAP, with the predominant phenotype being neuronal. In addition, 4% of the transplanted NSCs also expressed ChAT. At 3 weeks after birth, transplanted *Nmd^2J^* mice exhibited less muscle weakness and loss and were still able to pass the rotarod test at 5 weeks. Final survival was also shown to be longer. The loss of motoneurons and axons from L4 to L5 of the ventral nerve roots was also significantly reduced. In cell culture, the authors then demonstrated that the inhibition of GSK-3 and HGK kinase activation was a potential therapeutic factor that rescued Ighmbp2-deficient motoneurons after transplantation. In conclusion, based on the promising phenotypic improvements in the SMARD1 mouse, stem cell therapy approaches, or a combination of cell transplantation [61] and drug or gene therapy, could be considered for SMARD1 patients in the long-term, especially if gene therapy approaches do not have the desired effect, which will be discussed in the next chapter.

## 6. Gene Therapy in Mouse Models

In a recent study by Delgado and colleagues, it was very impressively demonstrated that induced neurons (iN) from compound heterozygous SMARD1 patients were shown to have shorter axons, fewer TuJ1+ somas, and more TuJ1+ neurons without neurites. AAV9-mediated overexpression of IGHMBP2 restored the neurite length of neurons in all disease cell lines, albeit to varying degrees, with some lines responding better to treatment than others. This leads to the assumption that additional non-IGHMBP2-related pathways are resulting in motoneuron loss [62]. This partial compensation of the SMARD1 phenotype could also be observed in the relevant mouse models summarized in Table 1 and underlines, once again, how important the integration of the corresponding mouse models is for optimizing therapeutic approaches.

**Table 1:** Phenotype of SMARD1 and CMTS2 mouse models, both those untreated and those treated using AAV9-*IGHMBP2*.

Mouse Model	Phenotype of Untreated Mice	Phenotype after AAV9-*IGHMBP2* Application
***Ighmbp2*^nmd−2J^/J (*Nmd^2J^*)** Spontaneous point mutation in intron 4 (c.39A->G) in C57BL/6 background Cox et al., 1998 [33] Grohmann et al., 2004 [35]		**IV, 5 × 10^11^ vg per pub at P1** by Nizzardo et al., 2015 [63]
	20–30% remaining Ighmbp2	Two-fold increase in IGHMBP2 levels. Increased mean life span.
	Survival ranges from 6 to 10 weeks up to 11 months. Lower body weight.	Increased weight but not reaching wild-type level. Preservation of motor units and restored NMJ function.
	Motoneuron loss, loss of NMJ function. Affected muscle fibers.	Improved muscle fiber morphology. Neuromuscular function improved but did not reach wild-type levels.
	Cardiomyopathy is present in later stages of the disease.	Slightly improved cardiac hypertrophy.
***Ighmbp2*^nmd−2J^/J (*Nmd^2J^*)** Spontaneous point mutation in intron 4 (c.39A->G) in C57BL/6 background Cox et al., 1998 [33] Grohmann et al., 2004 [35]		**ICV, 1.25 × 10^11^ vg per pub at P2,3** by Shababi et al., 2016 [47] and 2018 [64]
	20–30% remaining Ighmbp2.	30% increase in IGHMBP2 levels at 30 days.
	Survival ranges from 6 to 10 weeks up to 11 months. Lower body weight.	Increased survival to 11–12 months. Increased body weight but not reaching wild-type levels.
	Motor unit loss and loss of NMJ function. Affected muscle fibers.	Rescued loss of motoneurons and motor axons and increased motor performance, but not reaching wild-type levels.
	Degeneration of the diaphragm.	Improved muscle pathology in hind limbs and diaphragm.
	Cardiomyopathy is present in later stages of the disease.	Improved cardiac pathology.
***Ighmbp2*^nmd−2J^/J (*Nmd^2J^*)** Spontaneous point mutation in intron 4 (c.39A->G) in C57BL/6 background Cox et al., 1998 [33] Grohmann et al., 2004 [35]		**IV, 1.25 × 10^11^ vg per pub at P2** by Shababi et al., 2018 [64]
	20–30% remaining Ighmbp2.	Lower IGHMBP2 levels than ICV-treated *Nmd^2J^*.
	Survival ranges from 6 to 10 weeks up to 11 months. Lower body weight.	Equal survival rate as with ICV-injected *Nmd^2J^*.
	Motor unit loss and loss of NMJ function.	No rescue of hind limb contracture and motor function. Gastrocnemius muscle and NMJ defects are not rescued.
	Degeneration of the diaphragm. Cardiomyopathy is present in the later stages of the disease.	Improved diaphragm and cardiac pathology comparable to ICV-injected *Nmd^2J^*.
**FVB/NJ-*Ighmbp2^nmd/nmd^*** Point mutation in intron 4 (c.39A->G) introduced by CRISPR/Cas9 in congenic FVB/NJ background Shababi et al., 2019 [48]		**Low-dose ICV, 1.25 × 10^11^ vg per pub at P2,** **High-dose ICV, 2.5 × 10^11^ vg per pub at P2,3** by Shababi et al., 2021 [65]
	Life span of 18–21 days.	Low dose: increased survival up to 80 days. High dose: increased survival beyond 100 days.
	Lower body weight.	Low dose: weight gain, but not reaching wild-type levels. High dose: weight gain, but not reaching wild-type levels.
	Severe muscle weakness in the hind limbs.	Low dose: Rotarod performances improved to wild-type levels. Grip strength improved, but not to wild type level. High dose: Rotarod performances improved to wild-type levels. Grip strength improved, but not to wild-type levels.
	Reduced muscle fiber areas.	High dose: Muscle fiber pathology improved, but not to wild-type levels.
	Reduced NMJ innervation.	High dose: NMJ innervation improved to wild-type levels.
	Reduced motoneuron number and area.	High dose: motoneuron number and area are improved to wild-type levels.
**FVB/NJ-*Ighmbp2*^D564N^** Missense mutation D564N introduced by CRISPR/Cas9 in FVB/NJ background Smith et al., 2022 [49]		**ICV 5 × 10^11^ vg per pub at P2** by Smith et al., 2022 [49]
	Ighmbp2 level is similar to the wild-type control.	Ighmbp2/IGHMBP2 level is similar to the wild-type control.
	Lifespan of 12–22 days. Lower body weight.	5- to 10-times increased survival rate and weight gain, but not reaching wild-type levels.
	Muscle weakness in the hindlimbs.	Improved motor function (time-to-right, grip strength, hind limb splay, grip strength, and rotarod), but not reaching wild-type levels.
	Reduced motoneuron numbers and size. Decreased number of innervated endplates.	Increased motoneuron number, but not reaching wild-type levels. Increased innervated endplates, reaching wild-type levels.
	Reduced muscle fiber size.	Increased muscle fiber size, but not reaching wild-type levels.
	Decreased respiratory rate under normoxic conditions.	Improved respiratory frequency and number of apneas and erratic breathing under normoxic and hypercapnia + hypoxia conditions, similar to wild-type levels.

### 6.1. AAV9-IGHMBP2 Application to the Nmd^2J^ Model

The first experiments on AAV9-*IGHMBP2* transfers into mouse and human tissues were performed in 2015 [63] (Figure 3). They provided preclinical proof of principle, exhibiting the high efficacy of *IGHMBP2* gene therapy in the *Nmd^2J^* mouse, as well as in SMARD1 iPS cells. They performed intravenous applications into the facial vein, with a virus concentration of 5 × 10^11^ viral genomes (vg) per mouse on P1. Four weeks later, the mice were examined for pathological features. According to the upregulation of the IGHMBP2 protein in the spinal cord, the AAV9 application protects motoneurons and axons, rescues the neuromuscular phenotype, and improves animal survival and myofiber size in skeletal muscles and the heart [63]. This is consistent with the observation that SMARD1 iPSC-derived motoneurons overexpressing IGHMBP2 exhibit significantly improved neuronal survival and cellular differentiation [63]. The very first description of AAV9-*IGHMBP2* applications in the *Nmd^2J^* mouse was followed by a study by Sahabi and colleagues [47] (Figure 3C and Table 1). IGHMBP2 was expressed under a chicken β-actin (CBA) promoter along an intron in the 5′ region and a synthetic poly A site. For phenotypical analyses, a single-stranded vector was applied intracerebroventricularly at a lower concentration (1.25 × 10^11^ vg) at P2 and 3 and a higher concentration (2.5 × 10^11^ vg) at P2, 3, 4. ICV injection was preferred because it results in stable peripheral transduction at neonatal time points due to the low blood–brain barrier. However, the higher vector dose resulted in an increased mortality rate at P19-22. In addition, both virus titers led to the development of hydrocephalus in some mice 4–6 weeks after injection. In conclusion, both survival and body weight were significantly improved in *Nmd^2J^* mice at the lower dose. Rotarod performance and grip strength measurements showed a significant improvement at both doses, but the treated *Nmd^2J^* mice did not reach the control levels. Muscle changes commonly observed in *Nmd^2J^* mice in the gastrocnemius, quadricep muscles, and the diaphragm—such as muscle mass, fiber size, centralized nuclei, and interstitial/periarterial fibrosis—were significantly improved after low-dose applications eight weeks after injection, but again, they did not reach control levels. To examine the improvement in the large-diameter motor axon, all axons within the fifth lumbar motor root (L5) were examined at 8 weeks. Treated *Nmd^2J^* mice exhibited 64% recovery relative to motor axons, compared to 47% in untreated mice. In addition, the number of motoneurons, the number of myelinated motor axons, and the innervation of the gastrocnemius muscles were also significantly improved, but they did not reach control levels. In conclusion, a general 30 to 50% improvement in pathological changes was observed in the *Nmd^2J^* mouse after treatment, with no negligible gender effects. This, in turn, corresponds to the approximate 30% increase in IGHMBP2 expression levels in the *Nmd^2J^* treated with a low dose of virus, indicating a clear gene dose effect.

In the following publication, Shababi and colleagues aimed to show that the efficiency of the therapy strongly depends on the administration route of AAV9-*IGHMBP2* [64] (Table 1). Therefore, they evaluated low-dose (1.25 × 10^11^ vg) ICV versus IV (via facial vein) applications in *Nmd^2J^* mice, with respect to phenotypic expression. In low-dose ICV-treated *Nmd^2J^* mice, the IGHMBP2 level in the spinal cord 12 days post-injection is significantly higher than in IV-treated mice, according to Western blot analyses. Based on this fact, the following observations were made. The survival rate in *Nmd^2J^* mice was comparable in both applications. However, weight gain in ICV-treated animals was already significantly better than in IV-treated animals. Rotarod, grip strength, and hind limb contracture studies were also significantly better in ICV-treated *Nmd^2J^* mice than in IV-treated animals but did not reach control levels. These observations were supported by studies of the gastrocnemius muscle and the neuromuscular junction. Virtually no improvements in muscle pathology and innervation were observed in intravenously injected *Nmd^2J^* mice. Surprisingly, these differences in viral delivery were not observed in the diaphragm and the heart; diaphragm and cardiomyocytes were similarly rescued. IV- and ICV-treated *Nmd^2J^* hearts also showed similar reductions in interstitial and periarterial fibrosis and cardiac dysfunction.

In conclusion, the low-dose intracerebroventricular administration of AAV9- *IGHMBP2* increases IGHMBP2 levels in the spinal cord significantly more than intravenous administration. This, in turn, leads to a significant improvement of the atrophic phenotype with ICV compared to the IV application, although no control level is reached. In contrast, the myopathic phenotype observed in the diaphragm and the heart of the *Nmd^2J^* mouse is equally improved by both applications. However, control levels were not reached.

### 6.2. AAV9-IGHMBP2 Application to the FVBIghmbp2^nmd/nmd^ Model

Based on the postulated modifying effect of a region on chromosome 13 of the *Nmd^2J^* mouse [33], Shababi and colleagues generated an *Nmd^2J^* mouse on an FVB/NJ background [48] (Table 1). The point mutation in intron 4 of the Ighmbp2 gene originally described in the *Nmd^2J^* mouse was inserted into an FVB background using CRISPR/Cas. This resulted in a shortened but stable lifespan (18–21 days) of FVB*Ighmbp2^nmd/nmd^* mice. In contrast, all atrophic disease patterns were comparable to the *Nmd^2J^* mouse. In a subsequent study, Shababi et al. then showed, very impressively, that at low and high doses, applications at an earlier time point (P2-3), as opposed to a later time point (P6–P8), had a significantly improved effect on survival, motor function, muscle pathology, gastrocnemius NMJ pathology, and spinal motoneuron numbers from L3 to L5 [65]. However, it must be acknowledged that the application of AAV9-*IGHMBP2* at a later time point also leads to a significant improvement of the disease pattern, but not as significant as for P2-3 [65].

### 6.3. AAV9-IGHMBP2 Application to a SMARD1 Mouse Model with Respiratory Distress

SMARD1 patients develop diaphragmatic paresis at a very early age and require mechanical ventilation (Table 1). This respiratory distress is very weak in the *Nmd^2J^* mouse, independent of the genetic background (Bl6/FVB). The mouse develops myopathy of the diaphragm [35,45] only at a later stage of the disease. To generate a mouse model that is representative of respiratory distress to investigate the AAV9-*IGHMBP2* application efficiency on the phenotype, Smith et al. used CRISPR/Cas to create mouse models with point mutations in the *Ighmbp2* gene [49] (Table 1). One of these models, FVB/NJ-*Ighmbp2*^D564N^*,* carries the D564N mutation (D565N in humans). The mutation is in exon 12 in motif V within domain 2A. The model is a homozygous mutant with unaltered Ighmbp2 levels and nicely mimics the typical phenotypic changes observed in SMARD1 patients and the *Nmd^2J^* mouse. It has an average survival of 16–17 days, develops hind limb muscle atrophy, and exhibits reduced motor function. The number and size of spinal motoneurons are reduced at P15. In addition, the innervation of the gastrocnemius muscle is also impaired (P7-15) and the muscle fibers are reduced in size. Crucially, however, respiratory distress develops in this SMARD1 model. Whole-body plethysmography was performed on SMARD1 mice on day 12. Respiratory frequency (breaths per minute), tidal volume (volume of air moved between normal inhalation and exhalation), minute ventilation (volume of gas inhaled from the lungs per minute) and mean inspiratory flow (amount of air inhaled at any time) were measured under normoxic and hypercapnia + hypoxia conditions. Under normoxic conditions, minute ventilation and mean inspiratory flow were the same between mutant and control. However, when challenged by applying hypercapnia and hypoxia conditions, the mutant did not respond by increasing ventilation and mean inspiratory flow in contrast to the wild-type mice. In contrast, the mutants exhibited a higher tidal volume, which was further increased under hypercapnia and hypoxia conditions. In addition, the mutants exhibited an increased number of apneas and erratic breathing. The administration of a higher dose of AAV9-*IGHMBP2* (5 × 10^11^ vg, injected ICV) improved respiratory frequency, erratic breathing, and tidal volume to wild-type levels under both conditions, which was observed together with a significantly improved motor phenotype. However, control levels were not reached. In conclusion, this mouse model provides a very good contribution to the investigation of therapeutic interventions in the SMARD1 animal model. However, as already shown in *Nmd^2J^*, AAV9-*IGHMBP2* applications, even during the early postnatal period, are not able to rescue the complete disease phenotype.

## 7. Conclusions

In recent years, mouse models have been used to gain comprehensive insight into potential therapeutic approaches against SMARD1. In particular, for AAV9 approaches, the knowledge of AAV9-*IGHMBP2* titers and delivery strategies (ICV or IV) formed the basis for applying AAV9-*IGHMBP2* therapies in SMARD1 patients. However, detailed analyses of AAV9-*IGHMBP2* applications in *Nmd^2J^*, FVB-*Nmd*, and FVB-*Ighmbp2^D564N^* mice suggest that the AAV9-*IGHMBP2* treatment does not result in the complete rescue of the disease phenotype, even when applied at the pre-symptomatic stage.

Therefore, it is now time to further investigate the disease mechanisms leading to SMARD1, which are still largely unknown. A better understanding of the cellular dysregulations in IGHMBP2-deficient iPSC-derived motoneurons or Ighmbp2-deficient primary mouse motoneurons will further advance the development of cell and stem cell therapies. These therapies could then be used in the future to complement and support AAV9 strategies for SMARD1 patients.

In addition, a better understanding of the disease mechanisms leading to SMARD1 will enable the development of new SMARD1-specific biomarkers. This, in turn, will allow a better and faster characterization of SMARD1 compared to other spinal muscular atrophies and could provide additional information about the disease status of the patients.
